# Mesenchymal stromal cells reduce evidence of lung injury in patients with ARDS

**DOI:** 10.1172/jci.insight.148983

**Published:** 2021-06-22

**Authors:** Katherine D. Wick, Aleksandra Leligdowicz, Hanjing Zhuo, Lorraine B. Ware, Michael A. Matthay

**Affiliations:** 1 Departments of Medicine and Anesthesia and; 2 Cardiovascular Research Institute, University of California, San Francisco, San Francisco, California, USA.; 3 Interdepartmental Division of Critical Care Medicine, University of Toronto, Toronto, Ontario, Canada.; 4 Division of Allergy, Pulmonary, and Critical Care Medicine, Department of Medicine, and; 5 Department of Pathology, Microbiology, and Immunology, Vanderbilt University Medical Center, Nashville, Tennessee, USA.

**Keywords:** Pulmonology, Stem cells, Cytokines, Endothelial cells, Respiration

## Abstract

**BACKGROUND:**

Whether airspace biomarkers add value to plasma biomarkers in studying acute respiratory distress syndrome (ARDS) is not well understood. Mesenchymal stromal cells (MSCs) are an investigational therapy for ARDS, and airspace biomarkers may provide mechanistic evidence for MSCs’ impact in patients with ARDS.

**METHODS:**

We carried out a nested cohort study within a phase 2a safety trial of treatment with allogeneic MSCs for moderate-to-severe ARDS. Nonbronchoscopic bronchoalveolar lavage and plasma samples were collected 48 hours after study drug infusion. Airspace and plasma biomarker concentrations were compared between the MSC (*n =* 17) and placebo (*n =* 10) treatment arms, and correlation between the two compartments was tested. Airspace biomarkers were also tested for associations with clinical and radiographic outcomes.

**RESULTS:**

Compared with placebo, MSC treatment significantly reduced airspace total protein, angiopoietin-2 (Ang-2), IL-6, and soluble TNF receptor-1 concentrations. Plasma biomarkers did not differ between groups. Each 10-fold increase in airspace Ang-2 was independently associated with 6.7 fewer days alive and free of mechanical ventilation (95% CI, –12.3 to –1.0, *P =* 0.023), and each 10-fold increase in airspace receptor for advanced glycation end-products (RAGE) was independently associated with a 6.6-point increase in day 3 radiographic assessment of lung edema score (95% CI, 2.4 to 10.8, *P =* 0.004).

**CONCLUSION:**

MSCs reduced biological evidence of lung injury in patients with ARDS. Biomarkers from the airspaces provide additional value for studying pathogenesis, treatment effects, and outcomes in ARDS.

**TRIAL REGISTRATION:**

ClinicalTrials.gov NCT02097641.

**FUNDING:**

National Heart, Lung, and Blood Institute.

## Graphical abstract

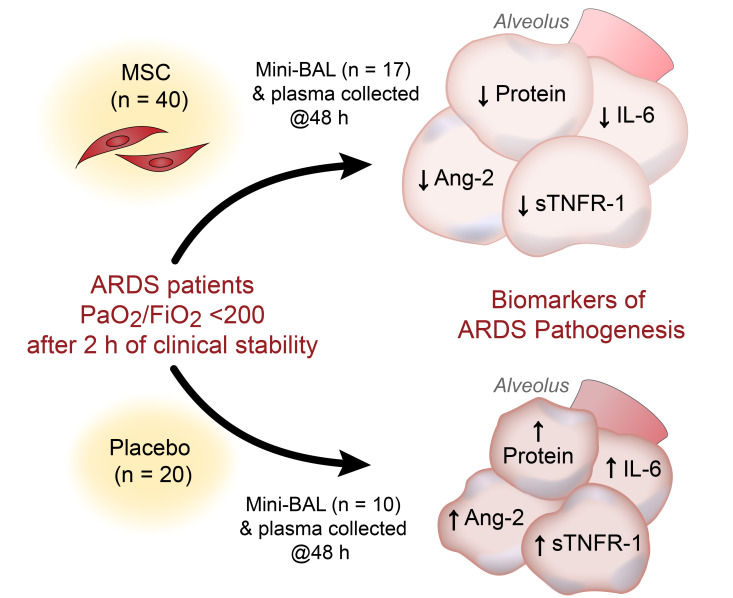

## Introduction

Studies of plasma biomarkers in the acute respiratory distress syndrome (ARDS) have improved understanding of the syndrome’s biologic heterogeneity (1) and differential treatment responses (2). Plasma biomarkers have also been used as biological endpoints in clinical trials of potential ARDS therapies (3–6). Airspace biomarkers are studied less frequently than plasma biomarkers, though they have been used as secondary endpoints in some clinical trials (7). Few studies have investigated simultaneous plasma and airspace biomarker measurements or the relationship between plasma and airspace biomarkers (8–10). To our knowledge, there has not been a systematic investigation of the relationship between plasma and airspace biomarkers or whether sampling the airspaces offers additional value in understanding ARDS pathogenesis, therapeutic interventions, and outcomes.

Airspace biomarkers could enhance the study of ARDS treatments by reflecting responses and possible therapeutic mechanisms in the lung better than circulating biomarkers. Mesenchymal stromal cells (MSCs) have been investigated as a novel therapeutic for ARDS because of their potential to act on many of the key pathways implicated in the pathogenesis of lung injury (11). MSCs promote a proresolving macrophage phenotype (12), enhance alveolar fluid clearance (13, 14), restore epithelial and endothelial barrier integrity (15), and reduce lung injury severity in preclinical models (16). The trial of treatment with allogeneic MSCs for moderate-to-severe ARDS (START trial, ClinicalTrials.gov NCT02097641) demonstrated the safety of MSCs in ARDS (17), but their efficacy in a clinical population has not definitively been proven. Because the primary therapeutic target in ARDS and one of the key determinants of the therapeutic potential of MSCs is the lung microenvironment (18–20), airspace samples could provide better evidence of biologic effect than samples collected from the peripheral circulation.

In this study, we analyzed plasma and airspace biologic samples from patients enrolled in the START trial (17). The first aim was to determine whether prespecified airspace biomarkers of endothelial injury (angiopoietin-2 [Ang-2]), inflammation (IL-6, IL-8, and soluble TNF receptor-1 [sTNFR-1]), and lung epithelial injury (receptor for advanced glycation end-products [RAGE]) collected 48 hours after therapeutic intervention differed between patients who received MSCs and those who received placebo. Total airspace protein concentration was also compared between patients in the MSC and placebo groups, as the concentration of total protein in the airspaces is a well-established biomarker of lung endothelial and epithelial protein permeability in experimental models of MSC therapy for ARDS (21, 22) and in ARDS clinical studies (23–25). The second aim was to study the relationship between 48-hour airspace and plasma biomarkers. The third aim was to determine whether airspace biomarkers are associated with prespecified clinical and radiographic outcomes.

## Results

### Patient characteristics.

**Figure 1:**
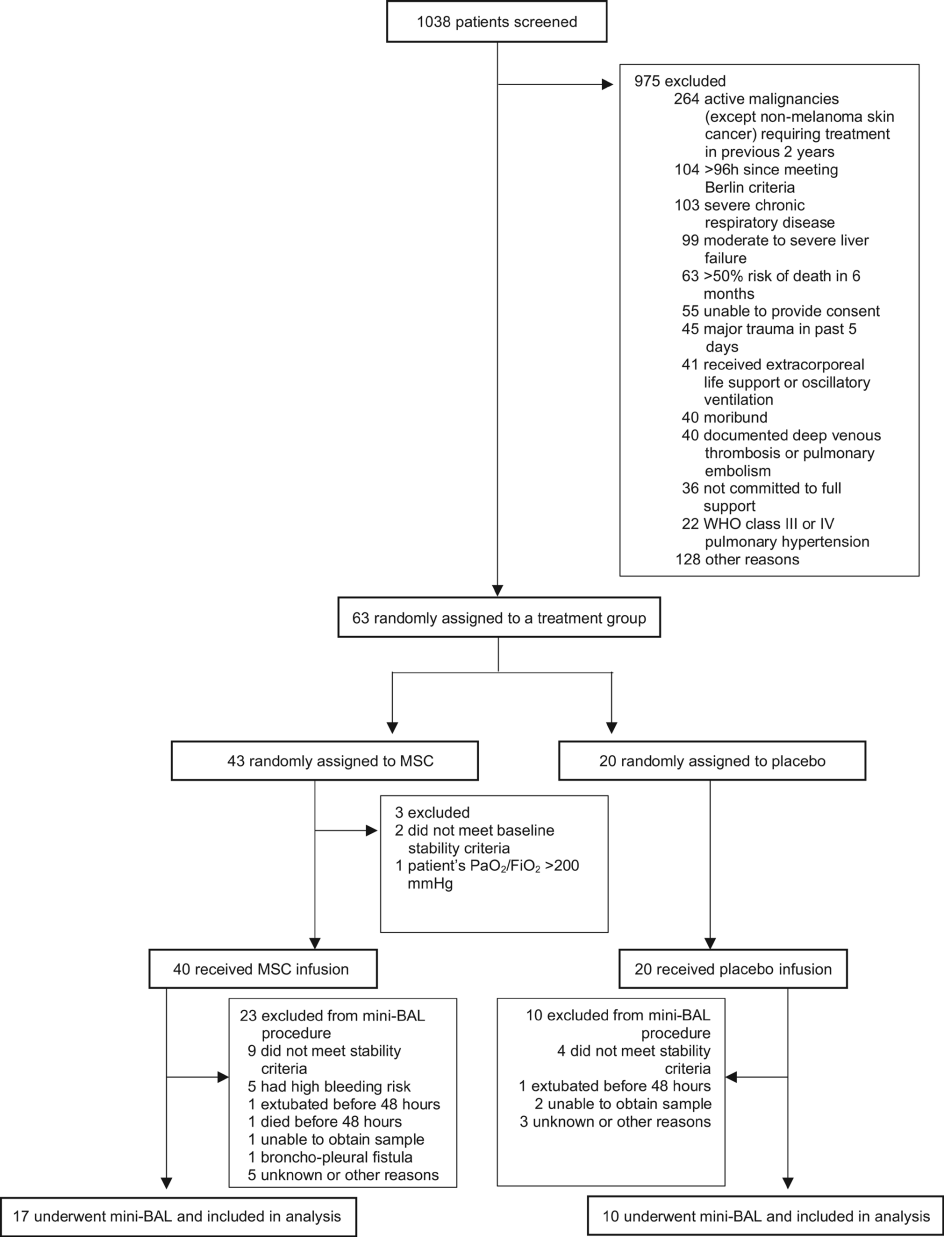
Study design.

A 48-hour airspace sample was available for 27 of the 60 (45%) patients (Figure 1). Patient demographics, ARDS risk factors, respiratory parameters on the day of nonbronchoscopic bronchoalveolar lavage (mini-BAL), and MSC viability did not significantly differ between patients who had a mini-BAL sample collected and those who did not (Supplemental Table 1; supplemental material available online with this article; https://doi.org/10.1172/jci.insight.148983DS1). Of the 27 patients who had a mini-BAL sample, there were no significant differences in baseline characteristics or respiratory variables at 48 hours between patients in the MSC group (*n =* 17) and those in the placebo group (*n =* 10; Table 1).

### Airspace biomarkers differed between MSC and placebo groups.

**Figure 2:**
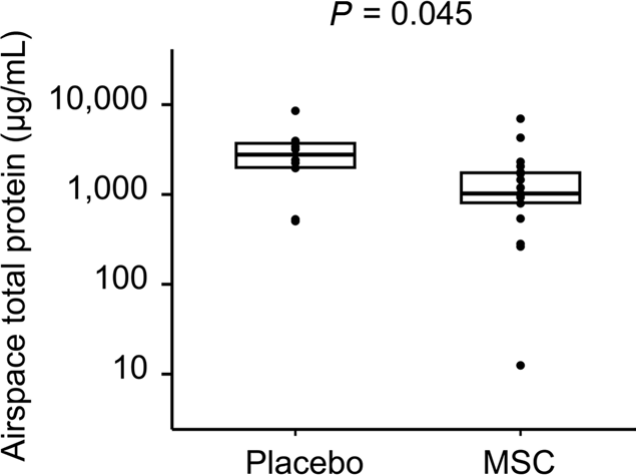
Airspace samples were collected 48 hours after treatment with either placebo or MSCs. Concentrations were not normally distributed after log_10_ transformation. Comparisons were made by Mann-Whitney *U* test on untransformed data. Horizontal lines and boxes represent median and IQR.

**Figure 3:**
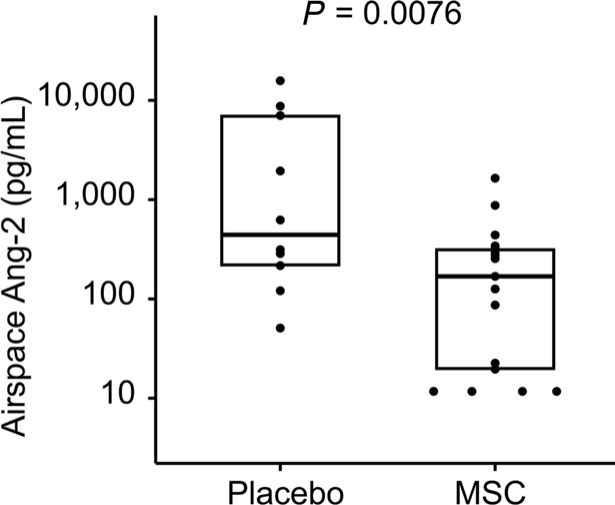
Airspace Ang-2 concentration in placebo and MSC groups. Airspace samples were collected 48 hours after treatment with either placebo or MSCs. Concentrations were normally distributed after log_10_ transformation. Comparisons were made by unpaired *t* test on transformed data. Horizontal lines and boxes represent median and IQR.

**Figure 4:**
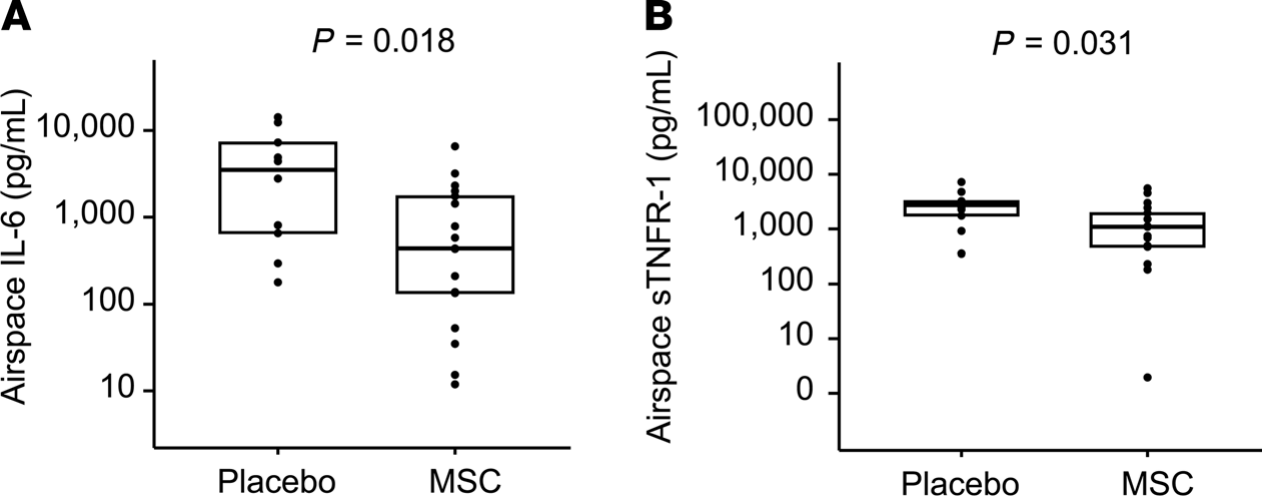
Airspace inflammatory biomarker concentrations in placebo and MSC groups. Airspace samples were collected 48 hours after treatment with either placebo or MSCs. Horizontal lines and boxes represent median and IQR. (**A**) IL-6. Concentrations were normally distributed after log_10_ transformation. Comparisons were made by unpaired *t* test on transformed data. (**B**) sTNFR-1. Concentrations were not normally distributed after log_10_ transformation. Comparisons were made by Mann-Whitney *U* test on untransformed data.

At 48 hours after treatment, all airspace biomarkers were numerically lower in the MSC arm relative to those in the placebo arm and most differences were statistically significant. Airspace total protein was significantly lower in the MSC group than in the placebo group (1025 μg/mL [IQR, 796–1770 μg/mL] vs. 2803 μg/mL [IQR, 1966–3761 μg/mL], *P =* 0.045), suggesting that MSC therapy reduced pulmonary vascular and epithelial permeability (Figure 2). Airspace total protein did not differ by primary ARDS risk factor (*P =* 0.16). Significantly lower airspace Ang-2 levels in MSC-treated patients compared with patients in the placebo group (169 pg/mL [IQR, 20–318 pg/mL] vs. 469 pg/mL [IQR, 217–7039 pg/mL], *P =* 0.0076) are consistent with less pulmonary endothelial injury in the MSC group (Figure 3). Two airspace biomarkers of inflammation, IL-6 and sTNFR-1, were significantly lower in the MSC group relative to the placebo group (435 pg/mL [IQR, 135–1743 pg/mL] vs. 3600 pg/mL [IQR, 652–7270 pg/mL], *P =* 0.018 and 1090 pg/mL [IQR, 470–1943 pg/mL] vs. 2740 pg/mL [IQR, 1745–3224 pg/mL], *P =* 0.031, respectively, Figure 4). There was a trend toward lower airspace IL-8 in the MSC group relative to the placebo group (5745 pg/mL [IQR, 771–10,435 pg/mL] vs. 12,624 pg/mL [IQR, 4114–23,879 pg/mL], *P =* 0.098). Airspace RAGE was numerically lower in the MSC group compared with that in the placebo group (1515 pg/mL [IQR, 565–5005 pg/mL] vs. 4006 pg/mL [IQR, 1191–11,866 pg/mL], but this difference did not reach significance (*P =* 0.19). There was no significant correlation between 48-hour airspace biomarker concentrations and MSC viability, which ranged from 46%–85% (Supplemental Figure 1 and Supplemental Table 2). In contrast to airspace biomarkers, none of the 48-hour plasma biomarkers differed between the 2 treatment groups (Supplemental Table 3).

One of the limitations of lavage procedures is variable return of instilled fluid, which could theoretically impact between-group differences in biomarker concentrations. Total sample volume was recorded for all but 1 sample (*n =* 26). Median mini-BAL sample volume was 4 ml (IQR, 2–4 ml) in the placebo group vs. 2 ml (IQR, 1.5–4 ml) in the MSC group (*P =* 0.41). Total volume ranged from 0.5 to 8 ml in the placebo group and 0.4 to 8 ml in the MSC group. We tested whether airspace biomarker concentrations correlated with total sample volume and found no significant correlations (Supplemental Table 4). In addition, total protein concentration was not associated with mini-BAL sample volume (ρ = –0.17, *P =* 0.40).

### Post hoc.

One of the samples in the MSC group was noted to have a considerably lower airspace total protein concentration than the other samples. Although there was no a priori reason to exclude this subject from our primary analysis, we performed a sensitivity analysis excluding this subject. A substantially lower airspace total protein concentration remained in the MSC group compared with placebo group, although the difference was no longer statistically significant (1107 μg/ml [IQR, 862–1914 μg/ml] vs. 2802 μg/ml [IQR, 1966–3761 μg/ml], *P =* 0.065). Other between-group differences remained statistically significant when this sample was excluded. Median airspace Ang-2 in the MSC arm was 212 pg/ml (IQR, 16–330 pg/ml) vs. 469 pg/ml (IQR, 217–7039 pg/ml), *P =* 0.012. Airspace IL-6 (506 pg/ml [IQR, 136–1871 pg/ml] vs. 3601 [IQR, 652–7270 pg/ml], *P =* 0.028) and sTNFR-1 (1104 pg/ml [IQR, 481–2186 pg/ml] vs. 2740 pg/ml [IQR, 1745–3224 pg/ml], *P =* 0.045) also remained statistically significantly lower in the MSC arm as compared with the placebo arm.

**Figure 5:**
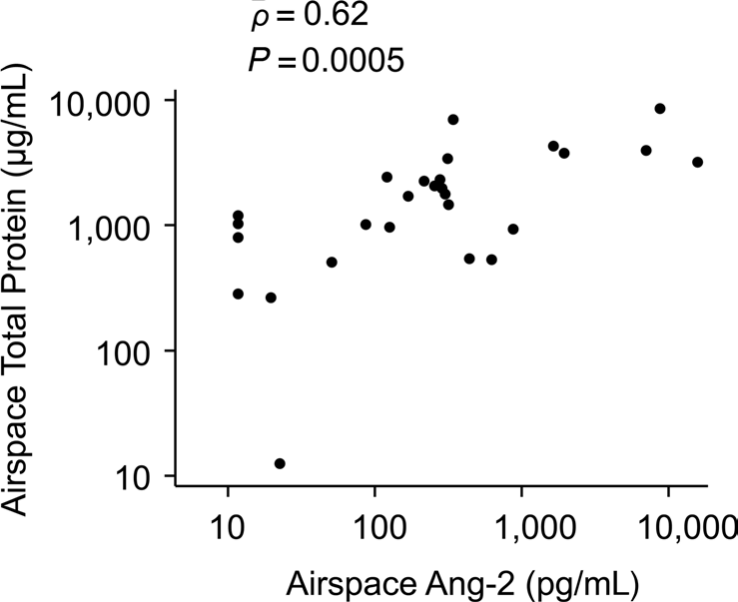
Correlation between airspace total protein and Ang-2 measured at 48 hours. Correlation coefficient depicts Spearman’s rho (ρ). Comparisons were made by Spearman’s rank correlation.

Since airspace levels of both total protein and Ang-2 were lower in MSC-treated patients compared with placebo controls, we tested the hypothesis that there would be a positive association between airspace Ang-2 and total protein. A statistically significant positive correlation between airspace Ang-2 and total protein was observed (ρ = 0.62, *P =* 0.0005; Figure 5). Alveolar permeability to protein is a result of both endothelial and epithelial barrier disruption. Although there was not a statistically significant difference in airspace RAGE levels between treatment groups, we tested the correlation between airspace total protein and RAGE given the biological plausibility of an association. There was a significant correlation between airspace RAGE and total protein (ρ = 0.42, *P =* 0.028).

### Airspace and plasma biomarker concentrations.

Airspace biomarker concentrations were significantly different from those in the plasma. IL-8, IL-6, and RAGE were more abundant in the airspaces, while sTNFR-1 and Ang-2 were more abundant in the plasma (Table 2). There was no significant correlation between airspace and plasma concentrations of IL-8 (ρ = –0.14, *P =* 0.48), sTNFR-1 (ρ = 0.013, *P =* 0.95), or Ang-2 (ρ = 0.18, *P =* 0.38). In contrast, there was a moderate correlation between airspace and plasma concentrations of IL-6 (ρ = 0.39, *P =* 0.04) and RAGE (ρ = 0.42, *P =* 0.03; Supplemental Figure 2).

### Airspace biomarkers and clinical outcomes.

Among airspace biomarkers and prespecified clinical, physiologic, and radiographic outcomes, there was an association between Ang-2 and ventilator-free days (VFDs) and between airspace RAGE and the day-3 radiographic assessment of lung edema (RALE) score. There were no other significant associations between biomarkers, including airspace total protein, and the prespecified ARDS outcomes. Higher airspace Ang-2 concentration was associated with fewer VFDs (ρ = –0.55, *P =* 0.019), while higher airspace RAGE concentration was associated with more radiographic pulmonary edema, specifically a higher day 3 RALE score (ρ = 0.64, *P =* 0.003). After adjustment for treatment arm and APACHE III, each 10-fold increase in airspace Ang-2 was independently associated with 6.7 fewer VFDs (95% CI, –12.3 to –1.0, *P =* 0.023), and each 10-fold increase in airspace RAGE was independently associated with a 6.6-point increase in day 3 RALE score (95% CI, 2.4 to 10.8, *P =* 0.004) (Table 3). In contrast, the 48-hour plasma Ang-2 was not significantly correlated with VFDs (ρ = 0.12, *P =* 1), and 48-hour plasma RAGE was not significantly correlated with day 3 RALE score (ρ = 0.33, *P* = 0.50).

## Discussion

There are two primary findings from this study. First, airspace biomarkers in patients in the START trial provide evidence of the biologic benefit of MSCs for reducing the severity of acute lung injury in patients with ARDS and bolster the rationale for continued testing of MSCs as therapy for ARDS. Second, the results support the value of sampling the distal airspaces in ARDS for investigating therapeutic interventions.

There were significantly lower values of airspace total protein, Ang-2, IL-6, and sTNFR1 in patients who received MSCs compared with those who received placebo. Airspace total protein and biomarker concentrations were not associated with total sample volume. These potentially novel findings recapitulate in a clinical population evidence of the therapeutic properties of MSCs that have been demonstrated in preclinical models (14, 16, 26, 27). In vitro, MSCs decrease alveolar type II (ATII) paracellular permeability to protein after injury with a mix of proinflammatory cytokines (28) and increase alveolar fluid clearance in an endotoxin-induced lung injury model in the ex vivo perfused human lung (14, 29). Preclinical experiments have also demonstrated that MSCs secrete paracrine factors with antiinflammatory effects and contribute to restoring endothelial and epithelial function (30–32).

We studied airspace total protein concentration as a key measure of alveolar endothelial and epithelial protein permeability. Airspace total protein has been investigated not only in preclinical models but also in clinical studies of patients with ARDS (23, 25, 33). Previous studies of patients with ARDS have demonstrated a correlation between airspace IgM, a large protein that is not abundant in the alveolar space in the absence of permeability pulmonary edema (34), and total protein (33). Additionally, airspace IgM and total protein provide similar prognostic information in patients with ARDS (33). We found no statistically significant difference in airspace total protein levels by clinical category of ARDS, suggesting that total protein concentration is a reasonable measure of alveolar permeability to protein even in the likely presence of neutrophil and bacterial infiltrates or other potential exogenous protein sources. In this study, the relationship between airspace Ang-2 and protein levels indicates that more severe lung endothelial injury is associated with higher alveolar protein permeability at 48 hours. The lower concentrations of both airspace Ang-2 and total protein in MSC-treated patients compared with patients who received placebo indicate that acute lung injury was attenuated by MSC therapy at the 48-hour time point. In contrast, there were no differences in plasma biomarkers between treatment groups. Therefore, airspace biomarkers may be more representative of pulmonary-specific therapeutic effects.

Preclinical studies support an initial localization of MSCs to the lung, offering further support for their putative therapeutic effect at the level of the pulmonary endothelium. Conceivably, the systemic administration of MSC therapy for nonpulmonary organ damage may be limited by first-pass trapping in the lung (35, 36). The mechanism of sequestration in the lung likely involves both molecular size and receptor-mediated interactions between MSCs and the endothelium via VCAM-1 (36). In the case of pulmonary injury, the tendency of MSCs to initially home to the pulmonary circulation could be advantageous. Although a biologic effect does not necessarily translate into clinical benefit, the results of this study provide a possible mechanistic rationale for further investigation of MSCs in clinical trials.

Different levels of biomarkers in the airspaces compared with the plasma underscore the importance of sampling the distal airspaces in the investigation of ARDS biology. For some biomarkers, there was no correlation between the pulmonary and systemic compartments. Thus, biomarkers detected in the airspaces versus the plasma likely reflect distinct biological processes. For example, IL-8 was markedly higher in mini-BAL fluid than in plasma, with no correlation between the two compartments. This suggests that IL-8 in the lungs and the blood may be derived from different cellular sources. Plasma IL-8 is a key biomarker in classifying and predicting outcomes from ARDS (37), but circulating levels of IL-8 do not specifically capture the lung inflammatory environment. Similarly, there was no correlation between airspace and plasma Ang-2 concentrations. The major source of Ang-2 is the endothelial Weibel-Palade body (38). Plasma Ang-2 concentration is higher in patients with indirect ARDS (e.g., nonpulmonary sepsis) than in those with direct ARDS (e.g., aspiration), likely because plasma Ang-2 reflects endothelial injury, not only at the level of the pulmonary microcirculation, but also the microcirculation of other organs (39). Thus, although plasma Ang-2 is important in sepsis-related ARDS (40), airspace Ang-2 concentrations may provide more direct insight into pulmonary endothelial injury. The results of this study indicate that sampling the blood is likely insufficient for reliably capturing airspace biology. Whether the pattern of airspace biomarkers differs by ARDS etiology, as is true for plasma biomarkers (39), and whether the relationship between airspace and plasma biomarkers is affected by the etiology of ARDS is an important avenue for future studies.

Plasma biomarkers, including RAGE, Ang-2, IL-8, and sTNFR-1, can predict the development of ARDS (41, 42) and ARDS outcomes (43, 44) and classify ARDS phenotypes that are durable over time (45, 46). The utility of airspace biomarkers in prognostic models has not previously been studied. We found that airspace Ang-2 not only reflects the biological response to MSC treatment, but also is associated with the important clinical outcome of VFDs. In contrast, plasma levels of Ang-2 drawn on the same day were not associated with VFDs. In addition, airspace RAGE was associated with the day 3 RALE score, a radiographic estimate of the severity of lung edema (47), while plasma RAGE from the same time point was not. Though airspace RAGE did not differ by treatment arm in this study, it may be a useful predictor of clinical outcomes in future research. Our results support further investigation of the prognostic value of airspace biomarkers alone or in combination with plasma biomarkers.

There are limitations to this study. First, the results are from a modest sample of patients from a clinical trial population that may not reflect larger unselected populations of patients with ARDS. Even though the sample was limited to 27 of the 60 enrolled patients, a substantial treatment effect was still observed. Aside from a higher proportion of abnormal coagulation tests in the MSC arm, reasons for exclusion between the 2 treatment groups were similar, including a similar proportion of patients excluded because of clinical instability. In addition, there were no significant differences between the patients who underwent the mini-BAL procedure and those who were excluded (Supplemental Table 1). Second, the mini-BAL procedure introduces an unknown dilution factor; however, the same saline volume of 40 ml was used in all mini-BAL procedures, and we found no significant correlation between any of the airspace biomarkers tested or airspace total protein and the lavage sample volume. Undiluted pulmonary edema fluid can only be obtained early in the course of ARDS, and all BAL experimental and clinical procedures require dilution from instilled saline. Even so, some biomarker concentrations were markedly higher in the airspaces than in the plasma (Table 2). Regardless of dilution, airspace samples are more specific to lung pathophysiology, since plasma biomarker concentrations also reflect contributions from other organs. Plasma biomarker levels may also be influenced by intravenous fluid administration, which is not standardized across patients (48). Regarding assessment of the MSC treatment effect, differences between treatment groups should not be confounded by the dilution factor. Third, cell counts were not obtained in lavage samples prior to processing, meaning that the effect of MSCs on inflammatory cell count could not be analyzed. In future studies, it would be valuable to test whether MSCs reduce airspace inflammatory cell infiltration. A reduction in airspace inflammatory biomarkers is not necessarily indicative of a reduction in immune cells or a reduction in immune cell function per se. However, these biomarkers reflect the overall proinflammatory environment of the lung in ARDS, which is complex and driven not only by infiltrating immune cells but also by soluble factors derived from the pulmonary epithelium and endothelium. Finally, airspace samples in our study were only collected at a single time point (48 hours after therapeutic intervention), because the study protocol required 2 hours of stable oxygenation and hemodynamics, which might have been disturbed by bronchoalveolar lavage during the baseline period. Therefore, we are unable to definitively conclude that MSC treatment lowered the airspace biomarkers from the time of enrollment, because a baseline mini-BAL fluid sample before MSC infusion could not be obtained. However, baseline characteristics, respiratory variables on the day of sample collection, and outcomes between the treatment arms were similar, and we would not expect a systematic difference in airspace biomarkers between patients who received MSCs and those who received placebo by chance alone. Additionally, samples were not collected beyond 48 hours, and therefore, we do not have evidence of the biologic effect of MSCs beyond 48 hours. In future studies, serial airspace samples, including baseline values, may provide valuable information.

In conclusion, the results of this study provide the first evidence to our knowledge that intravenous delivery of MSCs in patients with ARDS is associated with a reduction in alveolar permeability to protein, which may be mediated by a reduction in pulmonary endothelial and epithelial injury. Because samples were obtained 48 hours after MSC or placebo infusion, MSC therapy has a durable biological effect within this time frame. In addition, the study of airspace biomarkers provides substantial additional value to plasma biomarkers in understanding ARDS pathobiology, investigating novel treatments, and predicting outcomes. Inclusion of airspace sampling in future clinical trials may provide further insight into the biology and therapeutic targets of this complex critical illness syndrome.

## Methods

### Study design.

This is a cohort study nested within the START trial, a phase 2a multicenter double-blinded, randomized, controlled trial of intravenous MSCs for ARDS (17). In the START trial, 60 patients with ARDS and a ratio of partial arterial pressure of oxygen to fraction of inspired oxygen (PaO_2_/FiO_2_) of less than 200 mmHg were enrolled and randomized 2:1 to receive a single infusion of either 10 × 10^6^/kg predicted body weight MSCs or placebo. The proportion of viable (i.e., therapeutically active) MSCs administered was assessed post hoc using a trypan blue exclusion assay. Further details regarding trial design and patient characteristics are provided in Supplemental Methods and Supplemental Table 1.

### Biological sample collection, processing, and storage.

Blood samples were collected before study drug infusion and at 6, 24, and 48 hours after infusion. Airspace samples were obtained from a mini-BAL procedure performed 48 hours after infusion. We could not obtain baseline mini-BAL samples in this trial because the FDA required 2 hours of baseline respiratory and hemodynamic criteria before beginning the study product infusion. A mini-BAL procedure at baseline may have disturbed respiratory stability, thus delaying administration of the study product. Details of the mini-BAL procedure and exclusion criteria are provided in Supplemental Methods. Airspace and blood samples were centrifuged, and lavage supernatant and EDTA-anticoagulated plasma samples were cryopreserved at –80^o^C.

### Biomarker measurements.

IL-6, IL-8, sTNFR-1, Ang-2, and RAGE were measured in airspace samples collected 48 hours after study drug infusion. The same biomarkers were previously measured in plasma samples at baseline and 6, 24 and 48 hours after intervention. Biomarkers were measured by ELISA (R&D Systems). Airspace total protein was measured using a colorimetric assay (Thermo Fisher Scientific). Biomarkers below the limit of assay detection were assigned a value of one-half of the lowest point on the standard curve. All biomarker measurements were made without knowledge of the treatment group (i.e., MSC or placebo group).

### Clinical and radiographic outcomes.

Prespecified outcomes for this study were 28-day mortality, VFDs (defined as days alive and free from mechanical ventilation at 28 days after study enrollment, ref. 49), oxygenation index (OI; [FiO_2_ × mean airway pressure × 100]/PaO_2_) on day 3, change in OI between days 0 and day 3, ventilatory ratio ([minute ventilation × PaCO_2_]/[predicted body weight × 100 × 37.5]) on day 2, and RALE score (47) on day 3.

### Sensitivity analysis.

All patients who underwent mini-BAL were included in the primary analysis. It was noted post hoc, however, that 1 of the samples in the MSC arm had a considerably lower total protein concentration than the other samples. We therefore performed a post hoc sensitivity analysis of between-group differences in airspace biomarker concentrations excluding this sample using the same statistical methods as in the primary analysis.

### Statistics.

Descriptive analyses are presented as mean (SD), median (IQR), or number (percentage). If log_10_ transformation of nonnormally distributed variables yielded a normal distribution by the Shapiro-Wilk test, parametric statistical tests were performed on transformed values. If log_10_ transformation did not yield a normal distribution, nonparametric tests were performed on the original scale. Fisher’s exact test was used to compare categorical variables, unpaired 2-tailed *t* test or Mann-Whitney *U* test for between-group comparisons of continuous variables, 1-way ANOVA or Kruskal-Wallis test for multisample tests of variance, and paired 2-tailed *t* test or Wilcoxon’s signed rank test for within-group comparisons of continuous variables. Pairwise or rank correlation was used to test the relationship between airspace and plasma biomarkers, airspace sample volume and biomarker concentrations, biomarkers and MSC viability, and univariate relationships between airspace biomarkers and continuous clinical and physiologic outcomes.

If a significant correlation was observed between airspace biomarkers and a clinical or physiologic outcome, correlations between plasma biomarkers and the same outcome were also tested. Simple and multiple ordinary least-squares regression models were then tested. Model checking and diagnostics are provided in Supplemental Methods. Logistic regression was used to test single and multivariable relationships between airspace biomarkers and 28-day mortality. Individuals with missing outcome data were excluded from analysis. There were no missing data for VFDs. Two patients did not have a day 3 RALE score recorded. Two-sided *P* values of less than 0.05 were considered significant. A Bonferroni correction was used in multiple comparisons. All analyses were performed using Stata, version 16.1 (StataCorp).

### Study approval.

The institutional review boards at each trial site (University of San Francisco; Stanford University, Palo Alto, California, USA; University of Pittsburgh Medical Center, Pittsburgh, Pennsylvania, USA; Ohio State University, Columbus, Ohio, USA; Massachusetts General Hospital, Boston, Massachusetts, USA), the Food and Drug Administration (15331), and the National Heart, Lung, and Blood Institute (U01HL108713) approved the trial. Informed consent was obtained from all patients or an authorized surrogate, including for measurement of plasma and mini-BAL biomarkers.

## Author contributions

KDW, AL, MAM, and LBW conceived of and designed the study. KDW performed experiments. KDW, AL, and HZ analyzed data. KDW, AL, HZ, and MAM interpreted results of experiments. KDW drafted the manuscript. KDW, AL, MAM, and LBW edited and revised the manuscript. All authors approved final version of the manuscript.

## Supplementary Material

Supplemental data

Trial reporting checklists

ICMJE disclosure forms

## Figures and Tables

**Table 1:**
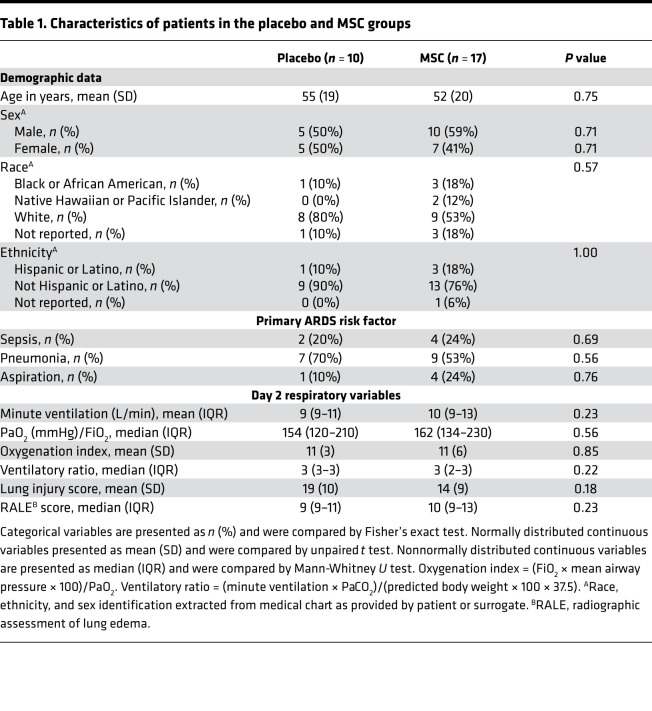
Characteristics of patients in the placebo and MSC groups

**Table 2:**
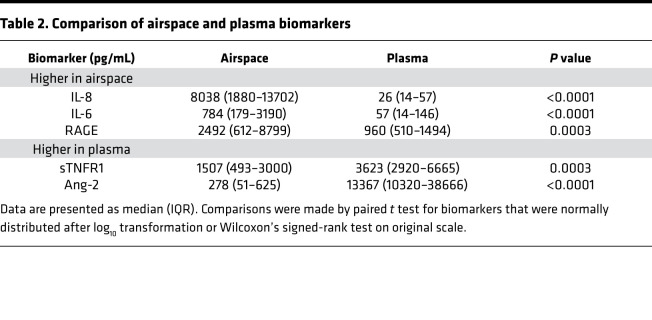
Comparison of airspace and plasma biomarkers

**Table 3:**
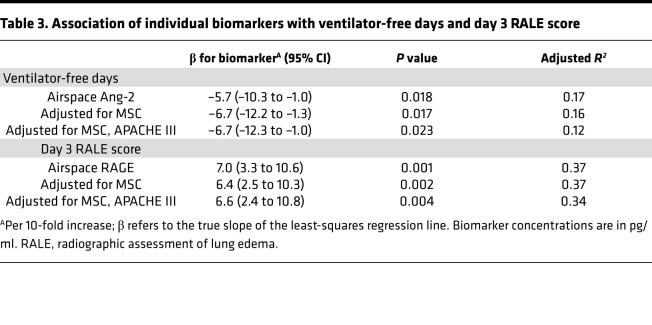
Association of individual biomarkers with ventilator-free days and day 3 RALE score

## Version 1 05/11/2021

In-Press Preview

## Version 2 06/22/2021

Electronic publication
