# Supplementary material for: Mesenchymal stromal cells reduce evidence of lung injury in patients with ARDS
Source: JCI Insight. 2021 Jun 22;6(12):e148983. doi: 10.1172/jci.insight.148983 (PMC8262503; doi:10.1172/jci.insight.148983)
Supplement: Supplemental data [file jciinsight-6-148983-s227.pdf]

Mesenchymal Stromal Cells Reduce Evidence of Lung Injury in Patients with ARDS

Online supplement

SUPPLEMENTARY METHODS:

START Trial Design and Participants

Patients were eligible for the trial of treatment with allogenic mesenchymal stromal cells (MSCs) for moderate to severe acute respiratory distress syndrome (START study) (1) if they were endotracheally intubated, had bilateral pulmonary infiltrates on chest imaging, a ratio of partial arterial pressure of oxygen to fraction of inspired oxygen (PaO_2/FiO_2) < 200 mmHg, were mechanically ventilated with a positive end-expiratory pressure (PEEP) of ≤ 8 cm H_2O (or ≤ 5 cm H_2O if there was evidence of barotrauma), and had no evidence of left ventricular failure or volume overload as the primary cause of pulmonary edema. Full exclusion criteria are available with the full text of the trial and its appendix (1) and included age < 18 years, ARDS present for >96 hours, major trauma within the past 5 days, not being committed to full support, an underlying medical condition with life expectancy < 6 months, severe underlying chronic lung disease, and partial arterial pressure of carbon dioxide ($PaCO_2$) > 50 mmHg. Participants were enrolled between March 24, 2014 and February 9, 2017.

Mini-BAL Procedure

The START study protocol included a non-bronchoscopic bronchoalveolar lavage (mini-BAL) procedure 48 hours after study drug infusion. A catheter in a protective sheath was inserted into the endotracheal tube through a standard bronchoscopy adapter and wedged blindly into the distal airway. 40 ml normal saline was instilled and then aspirated with an expected return of 5-10 ml. Exclusion criteria for this portion of the study included FiO_2 > 0.7 with positive end expiratory pressure (PEEP) > 14 cm H_2O, hemodynamic instability, open external ventricular

device or intracranial pressure > 15 mmHg or unstable, most recent international normalized ratio > 2.0 or prothrombin time 40, or most recent platelets < 50 x 10^3/mm^3.

Regression modeling and diagnostics

For linear regression, biomarkers were log_{10} transformed to better fit model assumptions and improve interpretability. Covariates included in the multivariable models were treatment arm (MSC vs. placebo) and severity of illness (Acute Physiology and Chronic Health Evaluation III [APACHE III] score). Checks for assumptions of linearity in single variable models were performed using smoothing of airspace and plasma biomarkers. Residual-versus-predicted plots were used to test linearity assumptions of multivariable models. Normality of residuals was checked using the Shapiro-Wilk test. Influential outliers were identified using DFBETA statistics. Excluding outliers did not substantively change the results of the models, and all observations are reported.

SUPPLEMENTARY TABLES

Supplementary Table 1. Characteristics of patients who did not vs. did have mini-BAL sample.

	No mini-BAL (n = 33)	Mini-BAL (n = 27)	p-value
**Patient Demographics**			
**Age**	56 (16)	53 (20)	0.56
**Gender**†			
Male	18 (55%)	15 (56%)	1.00
Female	15 (45%)	12 (44%)	
**Race**†			0.16
Asian	4 (12%)	0 (0%)	
Black or African American	1 (3%)	4 (15%)	
Native Hawaiian or Pacific Islander	1 (3%)	2 (7%)	
White	23 (70%)	17 (63%)	
Not reported	4 (12%)	4 (15%)	
**Ethnicity**†			0.16
Hispanic or Latino	2 (6%)	4 (15%)	
Not Hispanic or Latino	25 (76%)	22 (81%)	
Not reported	6 (18%)	1 (4%)	
**Primary ARDS Risk Factor**			
**Sepsis**	5 (15%)	6 (22%)	0.62
**Pneumonia**	18 (55%)	16 (59%)	0.94
**Aspiration**	12 (36%)	5 (19%)	0.31
**Day 2 Respiratory Variables**			
**Minute ventilation (L/min)**	10 (8-14)	10 (9-11)	0.82
**PaO_2/FiO_2**	155 (116-197)	154 (123-225)	0.36
**Oxygenation index**	12 (10-16)	12 (7-14)	0.27
**Ventilatory ratio**	2 (2-3)	2 (2-2)	0.98
**Lung injury score**	3 (3-4)	3 (2-3)	0.20
**RALE score**	13 (7-20)	16 (7-23)	0.21
**MSC viability (%)**	61 (15)	64 (12)	0.49

Categorical variables are presented as n (%), compared by Fisher's exact test. Normally distributed continuous variables presented as mean (SD), compared by unpaired t-test. Non-normally distributed continuous variables presented as median (IQR) compared by Mann-Whitney U test. Oxygenation Index = (FiO_2 x Mean Airway Pressure x 100)/PaO_2. Ventilatory ratio = (Minute ventilation x $PaCO_2$)/(Predicted body weight x 100 x 37.5). RALE: radiographic assessment of lung edema. †Race, ethnicity, and gender identification extracted from medical chart as provided by patient or surrogate.

Supplementary Table 2. Correlation between airspace biomarkers and MSC viability

Biomarker	Correlation coefficient	p-value
**Total protein (μg/ml)**	0.26	0.30
**Ang-2 (pg/ml)**	-0.056	0.83
**IL-6 (pg/ml)**	0.16	0.53
**IL-8 (pg/ml)**	0.014	0.95
**sTNFR1 (pg/ml)**	0.061	0.81
**RAGE (pg/ml)**	-0.14	0.59

Pearson's *r* is shown for biomarkers that were normally distributed after $\log_{10}$ transformation. Spearman's rho (ρ) is shown for biomarkers that were not normally distributed after transformation. P-values shown are not adjusted for multiple comparisons.

Supplementary Table 3. Comparison of plasma biomarkers by treatment group.

Biomarker (pg/ml)	Placebo (n = 10)	MSC (n = 17)	p-value
**IL-6**	56 (11-146)	58 (19-104)	0.64
**Ang-2**	13596 (6762-23921)	13367 (11265-21635)	0.76
**IL-8**	18 (9-55)	38 (19-57)	0.68
**RAGE**	976 (476-1553)	801 (585-1480)	0.70
**sTNFR1**	3781 (3138-4382)	3600 (2920-6665)	0.84

Data presented as median (IQR). Comparisons made by unpaired t-test for biomarkers that were normally distributed after $\log_{10}$ transformation or Wilcoxon signed rank test on original scale.

Supplementary Table 4. Correlation of airspace biomarkers with mini-BAL sample volume

Biomarker	Spearman's ρ	p-value
**Total protein (μg/ml)**	-0.17	0.40
**Ang-2 (pg/ml)**	-0.086	0.68
**IL-6 (pg/ml)**	-0.21	0.31
**IL-8 (pg/ml)**	-0.15	0.46
**sTNFR1 (pg/ml)**	-0.26	0.19
**RAGE (pg/ml)**	-0.25	0.22

N = 26. Mini-BAL sample volume was not normally distributed, and Spearman's rho (ρ) is depicted. P-values shown are not adjusted for multiple comparisons.

Supplementary Figure 1: Airspace total protein (A) and Ang-2 (B) vs. MSC viability. Airspace total protein was not normally distributed after $\log_{10}$ transformation, and Spearman's rho (ρ) is depicted. Airspace Ang-2 was normally distributed after $\log_{10}$ transformation, and Pearson's r is depicted.

A **B**

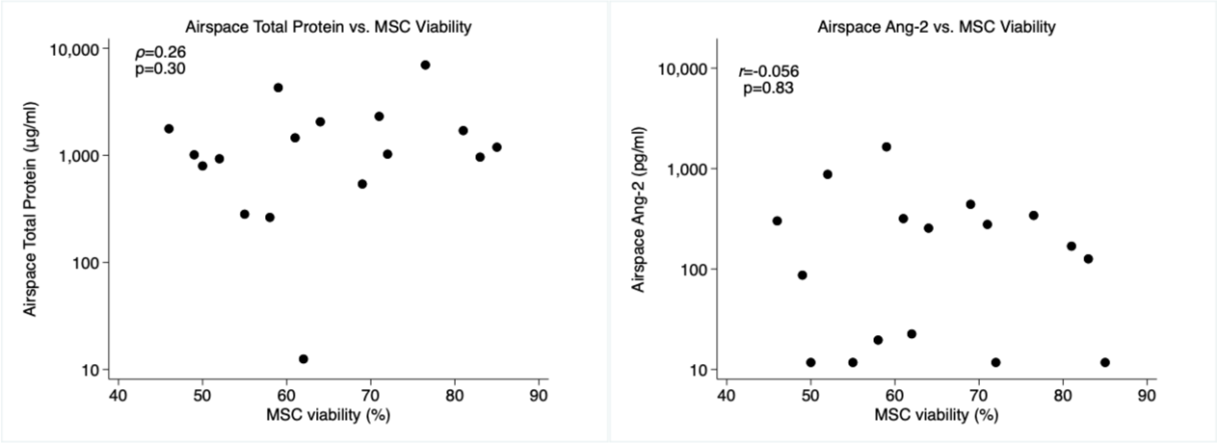

Supplementary Figure 2. Correlation between airspace and plasma biomarkers measured at 48 hours. Pearson's *r* is shown for biomarkers that were normally distributed after $\log_{10}$ transformation. Spearman's rho (ρ) is shown for biomarkers that were not normally distributed after transformation.

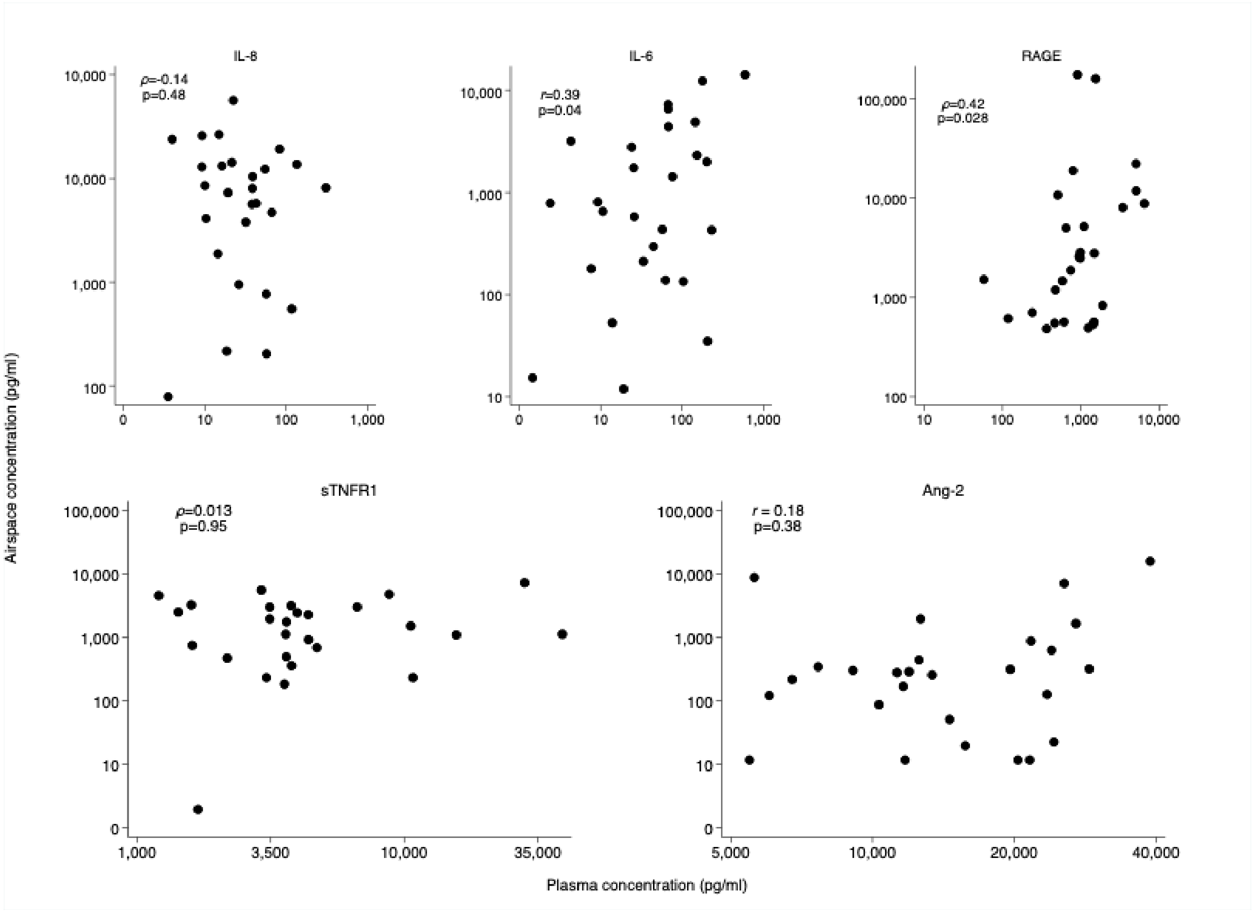

REFERENCES

1. Matthay MA, Calfee CS, Zhuo H, Thompson BT, Wilson JG, Levitt JE, Rogers AJ, Gotts JE, Wiener-Kronish JP, Bajwa EK, Donahoe MP, McVerry BJ, Ortiz LA, Exline M, Christman JW, Abbott J, Delucchi KL, Caballero L, McMillan M, McKenna DH, Liu KD. Treatment with allogeneic mesenchymal stromal cells for moderate to severe acute respiratory distress syndrome (START study): a randomised phase 2a safety trial. *Lancet Respir Med* 2019; 7: 154-162.
